# Prevalence of rickets-like bone deformities in rural Gambian children

**DOI:** 10.1016/j.bone.2015.04.011

**Published:** 2015-08

**Authors:** Helen L. Jones, Lamin Jammeh, Stephen Owens, Anthony J. Fulford, Sophie E. Moore, John M. Pettifor, Ann Prentice

**Affiliations:** a MRC Human Nutrition Research, Elsie Widdowson Laboratory, Cambridge, UK; b MRC Keneba, The Gambia; c Northumbria Healthcare NHS Foundation Trust, UK; d MRC International Nutrition Group, London School of Hygiene and Tropical Medicine, London, UK; e MRC/Wits Developmental Pathways for Health Research Unit, Department of Paediatrics, Faculty of Health Sciences, University of the Witwatersrand, Johannesburg, South Africa

**Keywords:** Africa, Calcium, Deformity, Rickets

## Abstract

The aim of this study was to estimate the burden of childhood rickets-like bone deformity in a rural region of West Africa where rickets has been reported in association with a low calcium intake. A population-based survey of children aged 0.5–17.9 years living in the province of West Kiang, The Gambia was conducted in 2007. 6221 children, 92% of those recorded in a recent census, were screened for physical signs of rickets by a trained survey team with clinical referral of suspected cases. Several objective measures were tested as potential screening tools. The prevalence of bone deformity in children < 18.0 years was 3.3%. The prevalence was greater in males (M = 4.3%, F = 2.3%, p < 0.001) and in children < 5.0 years (5.7%, M = 8.3%, F = 2.9%). Knock-knee was more common (58%) than bow-leg (31%) or windswept deformity (9%). Of the 196 examined clinically, 36 were confirmed to have a deformity outside normal variation (47% knock-knee, 53% bow-leg), resulting in more conservative prevalence estimates of bone deformity: 0.6% for children < 18.0 years (M = 0.9%, F = 0.2%), 1.5% for children < 5.0 years (M = 2.3%, F = 0.6%). Three of these children (9% of those with clinically-confirmed deformity, 0.05% of those screened) had active rickets on X-ray at the time of medical examination. This emphasises the difficulties in comparing prevalence estimates of rickets-like bone deformities from population surveys and clinic-based studies. Interpopliteal distance showed promise as an objective screening measure for bow-leg deformity. In conclusion, this population survey in a rural region of West Africa with a low calcium diet has demonstrated a significant burden of rickets-like bone deformity, whether based on physical signs under survey conditions or after clinical examination, especially in boys < 5.0 years.

## Highlights

• Nutritional rickets is reported in many parts of the world; calcium deficiency is suspected in some African and Asian countries.
• The burden of disease at the population level is largely unknown, especially in Africa.
• Prevalence estimates in the literature are highly dependent on the methods used.
• A 3.3% prevalence of rickets-like bone deformities was obtained in 6221 rural Gambian children aged < 18 years.
• Of those screened, 3 (0.05%) had active rickets at the time of medical examination.
• These prevalence rates were greater in children aged < 5 years (5.7%/1.5%) especially in boys (8.3%/2.3%).

## Introduction

Rickets is a childhood disorder of bone mineralisation at the growth plate, usually caused by inadequate concentrations of extra-cellular calcium or phosphate. The delay in or failure of endochondral ossification leads to deformation of the growth plate, the development of bone deformities and a reduction in linear growth [1,2]. Children with bone deformities may be severely disabled, have increased morbidity and decreased quality of life. The burden is currently greatest and the public health impact most substantial in developing countries, where crippling deformities reduce physical capacity and drain economic prospects [3]. Rickets is most commonly caused by vitamin D deficiency, although rickets in Sub-Saharan Africa, India and Bangladesh has been reported in children with a biochemical profile that does not suggest vitamin D deficiency but who may have calcium deficiency [2]. Prevalence estimates, however, are limited by the use of subjective assessments of deformity in large-scale surveys; there is a need to develop standard prediction tools for population screening [3–5].

We published a case series of rickets from The Gambia, West Africa, in which the aetiology was unknown but was associated with a very low calcium intake and elevated plasma fibroblast growth factor 23 (FGF23) [6]. The plasma 25-hydroxyvitamin D concentrations of these patients did not suggest vitamin D deficiency as a causal factor [6]. Rickets had not been formally described in The Gambia previously, a population typical of many Sub-Saharan African communities. The study reported here was designed to establish the prevalence and characteristics of rickets-like bone deformities in rural Gambian children aged 0.5–17.9 years in the province of West Kiang, which has been the focus of nutrition and health studies by the UK Medical Research Council for many years [6,7]. The survey was conducted using screening methods commonly employed in population surveys. In addition, five simple anthropometric measures (wrist width, wrist circumference, interpopliteal distance, intercondylar distance and intermalleolar distance) suitable for use by trained, non-medical staff were included to test their potential as screening tools. Suspected cases were referred for detailed clinical examination and diagnosis.

## Subjects and methods

West Kiang is a rural region of The Gambia, West Africa, latitude 13°N. The people are of Mandinka, Jola and Fula ethnicity and follow the Islamic faith, although purdah is not commonly practised. Undernutrition is prevalent and foetal, infant and child growth retardation is common. Most families are subsistence farmers, with men and women working the fields during the single rainy season (July–November). The diet is deficient in several micronutrients and calcium intakes are very low, averaging around 200 mg/d in children and 300–400 mg/d in women. Tropical sunshine is abundant throughout the year, and customary dress, although relatively conservative for women, does not restrict sunshine exposure of the face, arms and hands, and, in younger children, the lower legs and upper body [6,7]. Vitamin D status in this population is good, with plasma concentrations of 25-hydroxyvitamin D in children and adults well above those associated with vitamin D deficiency rickets [6–8].

The Rickets Prevalence Survey was conducted in the 30 villages and hamlets of West Kiang between February and October 2007. Sampling was framed within the 3-monthly West Kiang Demographic Surveillance System. All 6767 children who were alive, resident in West Kiang and aged ≥ 0.5 years and < 18.0 years on census day in February 2007 were eligible. The survey was approved by The Gambian Government/MRC Laboratories Joint Ethics Committee. Written informed consent was obtained from a parent or guardian of each child; all assessments were conducted with the implicit assent of the child.

The survey was explained to the villagers via a meeting with the village elders prior to the arrival of the survey team in each village. Each eligible child was traced, the family contacted and the survey explained by a member of the survey team.

Each participant was screened for bone deformities consistent with rickets by trained fieldstaff: knock-knee (genu valgum), bow-leg (genu varum) and windswept deformity of the lower limbs, ribcage deformities, bossing of the skull, and enlargement of the wrists, ankles and costochondral junctions. A brief questionnaire was completed to record if the child had difficulty in walking and running, bone pain at rest and during activity, and a greater tendency to fall than their peers. Prior to the survey, the survey team of six experienced community fieldworkers of the Medical Research Council were trained by a paediatric nurse (HLJ) to recognise rickets-like bone deformities and were provided with a photographic aide-memoire. They were also instructed in standardised anthropometry and the importance of regular equipment calibration.

A portable stadiometer (Leicester Height Measure, SECA, Hamburg, Germany) was used to measure the height of children ≥ 2.0 years. The child, with no shoes or head-dress, was positioned upright with a horizontal Frankfort plane. Feet were positioned together, parallel and flat on the floor. In children < 2.0 years, length was measured using a length board (Kiddimeter 100, Raven Equipment, Essex, England). The child, lightly clothed, was laid supine on the board with a vertical Frankfort plane. The spine and legs were kept straight with toes pointing upwards.

Wrist width was measured at the ulnar styloid using a Vernier caliper (model 675037, Silverline, Yeovil, England) with the forearm positioned lightly away from the body, fingers outstretched and palm facing downwards. Wrist circumference was measured using a small paper insertion tape (TALC, St Albans, England) by locating the ulnar styloid and encircling the radial margin at the widest part of the wrist.

A short, solid ruler held parallel to the floor was used to measure the distance between the legs at various points. The child was asked to remove or lift up clothing that would obscure the anatomical landmarks, and to stand upright, facing forward, with ankles or knees together, and feet parallel and flat on the floor. If this posed difficulties, the feet were positioned as close together as possible while keeping them parallel and flat on the floor. Interpopliteal distance (IPD) was defined as the distance between the two tendons of the semi-membranous muscle on each leg at the level of the popliteal fossa when in the standing position. Intercondylar distance (ICD) was defined as the distance between the two medial tibial condyles with ankles together. Intermalleolar distance (IMD) was defined as the distance between the two medial malleoli with knees touching. The anatomical landmarks were located by palpation and marked prior to the measurement. All leg measurements were obtained with the child standing.

Suspected cases of rickets were referred to the MRC Keneba Clinic for medical examination by one of two clinicians and, when clinically appropriate, for radiography of the wrists and knees and blood sampling for the measurement of total alkaline phosphatase. All radiographs were scored using the Thacher Radiographic Scoring Method [9] and reviewed by a consultant paediatrician (JP) experienced in the diagnosis and scoring of rickets. Total alkaline phosphatase activity was measured in plasma anticoagulated with lithium heparin by Kone Analyser 20i, Finland using the Kone Lab Alkaline Phosphatase (IFCC) kit. For the purposes of the survey, active rickets was defined as bone deformity with a Thacher Score > 1.5, an alkaline phosphatase > 960 U/l, or both.

Relative risks, calculated using MedCalc Software v12.7.8 are presented as RR [95%CI]. To account for the wide age range being surveyed, height (or length if < 2.0 years) was expressed as height-for-age SD-score (SDS) relative to the British Growth Reference [10]. Linear Model software was used for age and sex adjustment of height-SDS, IPD and wrist measures. Logistic regression and Receiver Operating Characteristic (ROC) curve analysis were used to assess the probability of a screening measure correctly identifying a clinically-confirmed lower limb deformity (DataDesk version 6.3, Data Description Inc; IBM SPSS version 22).

## Results

**Fig. 1:**
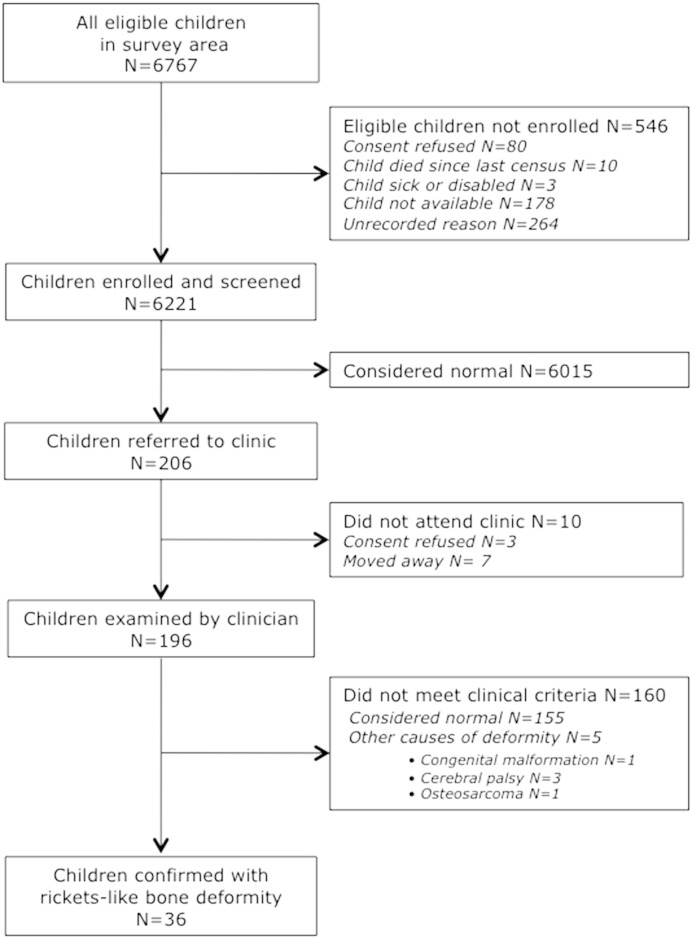
Flow chart of the Gambian survey of rickets-like bone deformities.

The results are given in Fig. 1. Of the 6767 children aged 0.5–17.9 years living in West Kiang and eligible for the survey, 6221 (92%), 3260 boys (52%) and 2961 girls (48%), were enrolled and screened for signs of rickets-like bone deformities. The median [IQR] age was 8.2 [4.3–12.1] years and was not significantly different between boys and girls; 1777 (28%) were < 5.0 years.

The survey team referred 206 children (3.3% of those screened) for clinical examination (Table 1). Of these, 58% were suspected of knock-knee, 31% bow-leg, 9% windswept deformity. Growth plate enlargement of the wrists, ankles or costochondral junctions was suspected in 2 other children (1% of those referred). No child was suspected of cranial bossing or rib-cage deformity. There were twice as many boys referred than girls referred (139 of 3260 (4.3%) vs 67 of 2961 (2.3%), RR = 1.9 [1.5–2.6] p < 0.001). A greater proportion of children aged < 5.0 years were referred than older children: 101 of 1777 (5.7%) and 105 of 4444 (2.4%) respectively, RR = 2.5 [1.9–3.2], p < 0.001 (Table 2).

Of the 206 children invited to the clinic for medical review, 196 attended. Of these, 36 children (0.6% of those screened, 18% of those reviewed) had a deformity considered by the clinician to be outside the normal range and consistent with rickets; 47% knock-knee, 53% bow-leg (Table 1). None was considered to have growth plate enlargement. The majority of cases of clinically-confirmed deformity were boys (83%), and boys appeared to be more likely to have bow-leg than girls, although the difference was not statistically significant (18 of 30 (60%) compared to 1 of 6 (17%), RR = 3.6 [0.6–22.1], p = 0.2).

The median [IQR] age of those with clinically-confirmed deformity was 3.6 [2.1–7.4]y: 72% were < 5.0 years old (Table 2). The prevalence of rickets-like bone deformity in the population of children < 5.0 years was 1.5% compared with 0.2% in older children (RR = 9.1 [4.4–18.8], p < 0.001), and was greatest in boys < 5 years (2.3%, Table 2). In the younger age group, knock-knee was more common than bow-leg (17 of the 26 cases (65%)) but bow-leg was more common in older children (8 of 10 cases (80%)).

Radiographs were obtained for 33 of the 36 children with clinically-confirmed rickets-like deformity. Of these, 3 children (9% of those X-rayed, 0.05% of those screened) had a Thacher score > 1.5 and were classified as having active rickets at the time of the medical examination; two of these, a brother and sister, were subsequently diagnosed with hereditary hypophosphataemic rickets with hypercalciuria as a result of mutation in the SLC34A3 sodium phosphate cotransporter gene [6,11]. Of the 30 children with a Thacher score ≤ 1.5, none had a total alkaline phosphatase activity > 960 U/l and were thus classified as non-active rickets-like deformities; radiographs of two of these children, aged 2 years and 4 years, showed the characteristic beaking of Blount disease [12].

Children identified at screening as phenotypically normal were short for their age compared with British children (height-for-age SDS: M = − 0.59 (SD 1.66); F = − 0.35 (SD 1.78)), in line with previous anthropometric studies in the region [13]. As expected because of their leg deformity, children with lower limb deformities were shorter than their peers (difference in height SDS after age and sex adjustment = − 0.60 (SE 0.27), p = 0.03), whereas those referred but considered to have a normal phenotype by a clinician were not (− 0.13 (SE 0.13), p = 0.3).

Of the screening measures trialled, only IPD reliably predicted clinically-confirmed rickets-like bone deformity, specifically bow-leg (logistic regression, P < 0.001; area under the ROC curve (SE, lower bound-upper bound) = 0.82 (0.07, 0.68–0.96), P < 0.001). IPD increased with age and was greater in boys. In linear models, IPD was a significant predictor of clinical deformity after age and sex adjustment (deformity versus normal phenotype = 21.1 ± SE 2.2 mm, p < 0.0001). The median [IQR] IPD values were: screened children considered normal = 65 [50–75] mm, n = 6015; clinically-confirmed cases = 110 [86–124] mm, n = 19. IMD was recorded as zero for 98% of screened children of normal phenotype but also for 35% of those with confirmed knock-knee. Likewise, 90% of children of normal phenotype and 11% of those with confirmed bow-leg had ICD recorded as zero at screening. These data suggest that it had been difficult during screening to position the legs correctly for children with deformity.

Among those with IMD > 0, the measured distance was greater in younger children, was similar in boys and girls, but did not separate children of normal phenotype and those with clinically-confirmed knock-knee (median [IQR] = 29 [13–43] mm, n = 104 and 35 [20–44] mm, n = 11 respectively). Among those with ICD > 0, the measured distance was greater in older children, in girls and in cases with bow-leg deformity (P < 0.001) (median [IQR]: normal phenotype = 25 [18–30] mm, n = 605, bow-leg cases = 60 [36–80] mm, n = 17, respectively).

Wrist circumference and wrist width increased with age and were greater in boys but did not distinguish children of normal phenotype (n = 6184) and clinically-confirmed cases of leg deformity (n = 36) (median [IQR]: wrist circumference = 126 [112–140] mm and 114 [107–143] mm, respectively; wrist width = 41 [35–47] mm and 36 [33–44] mm, respectively). In linear models, wrist circumference was a weakly significant predictor of clinically-confirmed bone deformity after age and sex adjustment (deformity versus normal phenotype: wrist circumference = 3.0 SE 1.5 mm p = 0.05) wrist width was not (0.7 mm SE 0.5 mm p = 0.2)

## Discussion

Rickets is a major public health problem, especially in developing countries and in some ethnic groups living in more affluent countries [2]. Vitamin D deficiency is the most common cause but calcium deficiency is implicated in African and Asian countries where vitamin D status, as measured by the plasma concentration of 25-hydroxyvitamin D, is above that typically associated with vitamin D deficiency rickets.

This survey of children living in a resource-poor, rural region of The Gambia, West Africa, recorded a prevalence of rickets-like bone deformity similar to that reported in Nigeria [14,15] and Bangladesh [16], where calcium deficiency is also a likely contributory factor (Table 3). These rates are lower than those of the Middle-East and Asia, where prevalence rates of 10–70% have been recorded [2,4]. This may partly reflect differences in aetiology, because vitamin D deficiency is common in those regions. However, it may also reflect the different methodologies used: some estimates were from population surveys based on visual appearance, occasionally with referral for radiography; others were from medical examination of children in hospital. The age groups screened also differed: some included only children < 3.0 years or < 5.0 years, others included all children and adolescents. Such methodological differences make comparisons difficult [3]. The results of the Gambian survey illustrate these problems. When calculated using screening information based on physical signs the prevalence of rickets-like bone deformities was five times greater than after medical examination, and the majority of children with rickets-like deformity confirmed by a clinician did not have active rickets. Similarly, the prevalence expressed as a proportion of children < 5.0 years was three times greater than that for all children < 18.0 years.

Some of these difficulties might be reduced by the use of objective measures in population surveys. In the Gambian survey, several simple anthropometric measures were evaluated as potential screening tools for knock-knee and bow-leg deformities. However, only IPD showed some promise for improving the identification of bow-leg by non-medical staff. In addition, because diagnostic radiographs and alkaline phosphatase measurements were only obtained on those children with bone deformities confirmed by a clinician, the sensitivities and negative predictive values of the screening and clinical assessments were unknown. The actual prevalence of active rickets may, therefore, have been underestimated.

The aetiology of rickets-like bone deformity in the Gambian population is unclear. In our case series of rickets patients, the biochemical profiles indicated urinary phosphate wasting associated with elevated plasma FGF23 and 1,25-dihydroxyvitamin D concentrations; vitamin D deficiency was discounted as causal factor [6]. A hypothesis was developed that linked the high circulating FGF23 to a chronically low calcium intake [6]. However, because low calcium intake is ubiquitous in this population and only relatively few children develop rickets-like bone deformities, additional factors must be involved. We have recently reported an inverse relationship between iron status and FGF23 in this population [8,17] that was accentuated in children with a family history of bone deformity [8]. This suggests that Gambian children with poor iron status and/or a genetic predisposition are at greater risk of developing rickets because of their low calcium intake, possibilities that will be explored in future studies.

In our survey, the prevalence of rickets-like deformity was greater in younger children than in older children, and greater at all ages than the prevalence of active rickets. Such findings have been reported in similar surveys elsewhere. Peak prevalence of rickets in Africa and Asia has generally been reported to occur at 3–5 years of age, occasionally with a second peak in adolescence [1]. Although longitudinal surveys have not been performed, this implies that the condition resolves over time in some individuals, possibly during periods when skeletal growth is comparatively slow. The bone modelling and remodelling required for the disappearance of a visible lower limb deformity takes many months or years compared to resolution of the metabolic perturbations and radiographic signs that define active rickets.

In summary, this survey of children < 18.0 years in West Kiang, The Gambia, demonstrated a prevalence of rickets-like bone deformity of 3.3% based on physical signs by trained survey staff, and 0.6% after medical examination by a clinician. These rates were greater for children < 5.0 years (5.3%/1.5%), especially in boys (8.3%/2.3%). These estimates reveal a significant burden of lower limb bone deformity in this region of rural Gambia, especially in children < 5.0 years, ranging from 1 in 19 children (1 in 12 boys) based on physical signs to 1 in 67 children (1 in 43 boys) based on medical examination.

## Disclosure statement

The authors have nothing to disclose.

## Figures and Tables

**Table 1:** Prevalence of rickets-like bone deformities in West Kiang, The Gambia: all ages 0.5–17.9 years.

	Referred after screening^a^			Confirmed after clinical review^b^			
	N	% Screened	% Referred	N	% Screened	% Reviewed	% Confirmed
Total	206	3.3	–	36	0.6	18	–
	(139, 67)	(4.3, 2.3)	–	(30, 6)	(0.9, 0.2)	(23, 10)	(83, 17)
Knock knee	119	1.9	58	17	0.3	9	47
	(71, 48)	(2.2, 1.6)	(51, 72)	(12, 5)	(0.4, 0.2)	(9, 8)	(40, 83)
Bow leg	63	1.0	31	19	0.3	10	53
	(51, 12)	(1.6, 0.4)	(37, 18)	(18, 1)	(0.6, 0.03)	(14, 2)	(60, 17)
Windswept	19	0.3	9	0	0	0	0
	(13, 6)	(0.4, 0.2)	(9, 9)	(0, 0)	(0, 0)	(0, 0)	(0, 0)
GPE^c^	2	0.03	1	0	0	0	0
	(1, 1)	(0.03, 0.03)	(1, 1)	(0, 0)	(0, 0)	(0, 0)	(0, 0)
Not recorded^d^	3	0.05	1	–	–	–	–
	(3, 0)	(0.09, 0)	(2, 0)				

Numbers are for both sexes together with those for boys and girls (M, F) below in parenthesis.

a Total screened = 6221, M = 3260, F = 2961.

b Total reviewed = 196, M = 132, F = 61. 10 children, all aged > 5.0 years, referred to the clinic were lost to follow-up (M = 7, F = 3).

c GPE = growth plate enlargement of wrists, ankles or costochondral junctions with no leg signs; 4 other children were suspected of GPE in addition to leg signs.

d Not recorded = physical sign not recorded at screening.

**Table 2:** Prevalence of rickets-like bone deformities in West Kiang, The Gambia: children aged < 5.0 years.

	Referred after screening^a^			Confirmed after clinical review^b^			
	N	% Screened	% Referred	N	% Screened	% Reviewed	% Confirmed
Total	101	5.7	–	26	1.5	26	–
	(76, 25)	(8.3, 2.9)	–	(21, 5)	(2.3, 0.6)	(28, 20)	
Knock knee	66	3.7	65	17	1.0	17	68
	(47, 19)	(5.2, 2.2)	(62, 76)	(12, 5)	(1.3, 0.6)	(16, 20)	(57, 100)
Bow leg	22	1.2	22	9	0.5	9	35
	(19, 3)	(2.1, 0.3)	(25, 12)	(9, 0)	(1.0, 0)	(12, 0)	(43, 0)
Windswept	10	0.6	10	0	0	0	0
	(7, 3)	(0.8, 0.3)	(9, 12)	(0, 0)	(0, 0)	(0, 0)	(0, 0)
GPE^c^	0	0	0	0	0	0	0
	(0, 0)	(0, 0)	(0, 0)	(0, 0)	(0, 0)	(0, 0)	(0, 0)
Not recorded^d^	3	0.2	3	–	–	–	–
	(3, 0)	(0.3, 0.0)	(4, 0)				

Numbers are for both sexes together with those for boys and girls (M, F) below in parenthesis.

a Total screened = 1777, M = 912, F = 865.

b Total reviewed = 101, M = 76, F = 25. All children aged < 5.0 years referred to the clinic attended for review.

c GPE = growth plate enlargement of wrists or ankles with no leg signs; 4 other children were suspected of GPE in addition to leg signs.

d Not recorded = physical sign not recorded at screening.

**Table 3:** Comparison of prevalence estimates from Africa and Asia of rickets-like deformity associated with calcium deficiency.

Country (region)	Survey	Age	Sample	Number	Method^a^	Prevalence %			Reference
	Date	(y)		Screened		All	Boys	Girls	
*All children and adolescents*									
The Gambia (West Kiang)	2007	< 18	Population	6221	PS	3.3	4.3	2.3	This paper
					PS + ME	0.6	0.9	0.2	
Bangladesh (National)	2008	< 16	Population	20,000	PS + ME	1.0	1.0	1.0	[16]
Bangladesh (Chittagong)	2008	< 16	Population	3249	PS + ME	2.2	–	–	[16]

*Younger children*									
The Gambia (West Kiang)	2007	< 5	Population	1777	PS	5.7	8.3	2.9	This paper
					PS + ME	1.5	2.3	0.6	
Nigeria (Jos)	1998	< 3	Community	218	PS	9.2	8.3	10.1	[14]
Nigeria (Jos)	2012	< 2	Community	647	PS + ME	1.2	–	–	[15]
Bangladesh (National)	2008	< 5	Population	7730	PS + ME	1.6	–	–	[16]
China (Shanxi Province)	2007	< 2	Population	250	PS	41.6	–	–	[4]
					PS + ME	3.7	–	–	

a PS = using physical signs only; PS + ME = using physical signs with medical examination of suspected cases.

## References

[1] Pettifor J.M., Glorieux F., Jueppner H., Pettifor J.M. (2012). Nutritional rickets. Pediatric bone — biology and diseases.

[2] Prentice A. (2013). Nutritional rickets around the world. J Steroid Biochem Mol Biol.

[3] Thacher T.D., Fischer P.R., Strand M.A., Pettifor J.M. (2006). Nutritional rickets around the world: causes and future directions. Ann Trop Paediatr.

[4] Strand M.A., Perry J., Jin M., Tracer D.P., Fischer P.R., Zhang P. (2007). Diagnosis of rickets and reassessment of prevalence among rural children in northern China. Pediatr Int.

[5] Thacher T.D., Fischer P.R., Pettifor J.M. (2002). The usefulness of clinical features to identify active rickets. Ann Trop Paediatr.

[6] Prentice A., Ceesay M., Nigdikar S., Allen S.J., Pettifor J.M. (2008). FGF23 is elevated in Gambian children with rickets. Bone.

[7] Prentice A. (2008). Vitamin D, deficiency: a global perspective. Nutr Rev.

[8] Braithwaite V., Jarjou L.M.A., Goldberg G.R., Prentice A. (2012). Iron status and fibroblast growth factor-23 in Gambian children. Bone.

[9] Thacher T.D., Fischer P.R., Pettifor J.M., Lawson J.O., Manaster B.J., James C. (2000). Radiographic scoring method for the assessment of severity of nutritional rickets. J Trop Pediatr.

[10] Freeman J.V., Cole T.J., Chinn S., Jones P.R.M., White E.M., Preece M.A. (1995). Cross-sectional stature and weight reference curves for the UK, 1990. Arch Dis Child.

[11] Braithwaite V., Pettifor J.M., Prentice A. (2013). Novel SLC34A3 mutation causing hereditary hypophosphataemic rickets with hypercalciuria in a Gambian family. Bone.

[12] Langenskiold A. (1989). Tibia vara. A critical review. Clin Orthop Relat Res.

[13] Dibba B., Prentice A., Ceesay M., Stirling D.M., Cole T.J., Poskitt E.M.E. (2000). Effect of calcium supplementation on bone mineral accretion in Gambian children accustomed to a low calcium diet. Am J Clin Nutr.

[14] Pfitzner M.A., Thacher T.D., Pettifor J.M., Zoakah A.I., Lawson J.O., Isichei C.O. (1998). Absence of vitamin D deficiency in young Nigerian children. J Pediatr.

[15] Thacher T.D., Fischer P.R., Isichei C.O., Zoakah A.I., Pettifor J.M. (2012). Prevention of nutritional rickets in Nigerian children with dietary calcium supplementation. Bone.

[16] ICDDRB (2009). National Rickets Survey in Bangladesh, 2008. Health Sci Bull.

[17] Braithwaite V., Prentice A.M., Doherty C., Prentice A. (2012). FGF23 is correlated with iron status but not with inflammation and decreases after iron supplementation: a supplementation study. Int J Pediatr Endocrinol.

